# Exon skipping-rich transcriptomes of animals reflect the significance of exon-shuffling in metazoan proteome evolution

**DOI:** 10.1186/s13062-019-0231-3

**Published:** 2019-01-16

**Authors:** Laszlo Patthy

**Affiliations:** Institute of Enzymology, Research Centre for Natural Sciences, Hungarian Academy of Sciences, Budapest, H-1117 Hungary

**Keywords:** Alternative splicing, Exon skipping, Exon-shuffling, Intron phase, Mobile domains, Symmetrical modules

## Abstract

**ᅟ:**

Animals are known to have higher rates of exon skipping than other eukaryotes. In a recent study, Grau-Bové et al. (Genome Biology 19:135, 2018) have used RNA-seq data across 65 eukaryotic species to investigate when and how this high prevalence of exon skipping evolved. They have found that bilaterian Metazoa have significantly increased exon skipping frequencies compared to all other eukaryotic groups and that exon skipping in nearly all animals, including non-bilaterians, is strongly enriched for frame-preserving events. The authors have hypothesized that “*the increase of exon skipping rates in animals followed a two-step process. First, exon skipping in early animals became enriched for frame-preserving events. Second, bilaterian ancestors dramatically increased their exon skipping frequencies, likely driven by the interplay between a shift in their genome architectures towards more exon definition and recruitment of frame-preserving exon skipping events to functionally diversify their cell-specific proteomes*.”

Here we offer a different explanation for the higher frequency of frame-preserving exon skipping in Metzoa than in all other eukaryotes. In our view these observations reflect the fact that the majority of multidomain proteins unique to metazoa and indispensable for metazoan type multicellularity were assembled by exon-shuffling from ‘symmetrical’ modules (i.e. modules flanked by introns of the same phase), whereas this type of protein evolution played a minor role in other groups of eukaryotes, including plants. The higher frequency of ‘symmetrical’ exons in Metazoan genomes provides an explanation for the enrichment for frame-preserving events since skipping or inclusion of ‘symmetrical’ modules during alternative splicing does not result in a reading-frame shift.

**Reviewers:**

This article was reviewed by Manuel Irimia, Ashish Lal and Erez Levanon. The reviewers were nominated by the Editorial Board.

## Background

The main forms of alternative splicing include the retention of introns and the exclusion of exons (exon skipping, ES) from the final transcripts. Intron retention is frequent in all eukaryotic groups, whereas animals are known to have significantly higher rates of exon skipping than other eukaryotes [1, 2]. In a recent paper Grau-Bové et al. [3] analyzed 65 eukaryotic species to investigate when and how animal transcriptomes shifted towards higher frequencies of exon skipping. Using datasets of transcriptomic and genomic data they have determined the frequency of exon skipping in all major eukaryotic lineages and have concluded that the frequency of exon skipping is significantly higher in animals than in all other eukaryotes. Comparison of the exon skipping frequencies of vertebrates, non-vertebrate bilaterians, non-bilaterians (cnidarians, poriferans, ctenophores and placozoa) and the closest unicellular relatives of metazoa revealed that bilaterians (and vertebrates in particular) have significantly higher exon skipping frequencies than their unicellular relatives or plants.

The authors also examined the relative frequency of exon skipping events that do not disrupt the reading frame (since the number of nucleotides of the skipped exon is divisible by 3). They have found that alternatively spliced exons of most animals were significantly enriched in 3n divisible lengths. A positive 3n bias was not observed in other eukaryotes, including plants, suggesting that 3n exon enrichment is an animal feature. Based on these data the authors have concluded that “*overall, the 3n bias in ES events recorded in animals suggests that the lengths of alternatively spliced exons are under selective pressure to avoid ORF disruptions, possibly due to an enrichment in functional protein isoform-producing ES events*”. The authors, however, did not provide an answer to the obvious question, why this selective pressure operates in multicellular organisms like animals but not in multicellular organisms like plants.

In the next sections of their paper Grau-Bové et al. [3] address the question of how exon skipping became more abundant in metazoa than in all other groups of eukaryotes. Since earlier studies have linked the level of alternative splicing to differences in the length of exons and introns, intron density, splicing site homogeneity etc., they have examined the influence of these factors on exon skipping by comparing exon skipping positive and negative exons and introns across the 65 eukaryotic species. These studies have identified a significant association between positive cases of exon skipping and weak 5′ and 3′ splice sites: exons with poorly defined intron–exon boundaries are more likely to be subject to exon skipping than those closer to the species consensus. A consistent association has also been found between exon skipping and shorter exon lengths and longer flanking introns: as a corollary there is a general positive relationship between exon skipping and higher intron-to-exon length ratios. These observations have led the authors to conclude that “*ES events across eukaryotes were globally associated with short exons flanked by longer introns, and with weak 5’ and 3’ splice sites. Inasmuch as these features are more common in animals and plants than in most eukaryotes, we can expect higher ES frequencies in these multicellular lineages*.” The authors, however, do not provide an explanation why the high frequency of exon skipping is characteristic of animals but not plants.

In summary, the paper of Grau-Bové et al. [3] has provided convincing evidence that animals (particularly vertebrates) have significantly higher ES frequencies than other eukaryotes, including plants and that all animals show high fractions of 3n exons among their ES events, but no such enrichment of 3n exons among ES events is observed in other eukaryotes, including plants. The paper, however, fails to explain why the pressure to maintain ORFs in the event of ES is an animal-specific trait or why the high frequency of exon skipping is characteristic of animals but not plants if the features facilitating ES events are common in animals and plants.

## Conclusions

Here we provide an explanation for the authors’ observations that also accounts for the difference between multicellular animals and multicellular plants.

In our earlier work we have suggested that the increased frequency of exon skipping in animals “*may be best explained by the intimate relationship between exon-shuffling and exon-skipping: novel ‘symmetrical’ (frame-preserving) exons generated by exon-duplication and exon insertion are particularly prone to exon-skipping. Since exon-shuffling played a major role only in the evolution of metazoa and became increasingly significant in the vertebrate lineage, it is not surprising that the frequency of exon-skipping reflects this trend*” [4]. According to this explanation the higher frequency of ES events in animals and the enrichment of 3n exons among ES events in animals reflect the fact that the majority of multidomain proteins unique to metazoa and indispensable for metazoan type multicellularity were assembled by exon-shuffling from ‘symmetrical’ (i.e. 3n) modules [5, 6], whereas this type of protein evolution played a minor role in other groups of eukaryotes, including plants [7–9]. Exon-shuffling continued to be a major source of evolutionary novelty during vertebrate evolution [8], explaining why this group of animals is characterized by the highest rate of frame-preserving exon skipping.

Since multidomain proteins constructed by exon duplication and exon shuffling have been assembled from ‘symmetrical exons’, skipping or inclusion of these exons leads to domain variants that represent different stages in the assembly process. In this sense, alternative splicing may reenact the assembly process. Accordingly, the high frequency of exon skipping in animals reflects the major significance of exon-shuffling in metazoan proteome evolution.

## Reviewers’ comments

### Reviewer’s report 1: Manuel Irimia, Centre de Regulació Genòmica, Barcelona Institute of Science and Technology and Universitat Pompeu Fabra (UPF), Spain.

## Reviewer’s comment.

As far as I understand it, this is not a regular review, since the MS directly discussed some of our recent work. In that sense, I guess I have an expected conflict of interest.

In this short piece, Patthy proposes that the higher frequency of frame-preserving exon skipping in Metazoa compared to all other eukaryotes that we have recently reported is due to the high frequency of exon-shuffling from ‘symmetrical’ modules that have generated a large amount of new genes in metazoans, but not in other lineages. This is certainly an interesting proposal that we have overlooked. However, there are a few uncertainties and caveats that would need to be addressed (here, or in a subsequent study). First, the literature cited in the present manuscript focuses on evidence of exon shuffling in multicellularity-related multidomain genes in bilaterians, since they were no other genomes available at the time of the studies (the latest is from 2005). Thus, it seems crucial to know whether the high frequency of exon-shuffling from ‘symmetrical’ modules occurred at the origin of bilaterians, metazoans or before. According to the author’s argument, this seems to have occurred by the origin of metazoans, which is also supported by more recent studies [1]. Second, there is therefore a gap between the evolutionary timing of exon shuffling and skipping-rich transcriptomes: high exon skipping seems to be a derived trait of bilaterian transcriptomes, whereas (i) ‘symmetrical’ exon shuffling (using exons in 1–1 phase) is also prevalent in the non-bilaterians *Trichoplax adhaerens* and *Nematostella vectensis* [1]; and (ii) the origin and diversification of many multicellularity-linked multidomain gene families is frequently older than bilaterians [2–4] and even animals [5–7]. Overall, this suggests that symmetrical exon shuffling (and its effects on the diversification of animal gene repertoires) coincides in time with the origin of the qualitative ‘3n bias’ in exon skipping, but not with quantitative increase in exon skipping. This means that this phenomenon is probably not linked to the quantitative differences between bilaterian animals (3n bias, high exon skipping, and affected by shuffling), non-bilaterians (3n bias, lower skipping rates, and affected by shuffling) and plants (no 3n bias, lower skipping rate, and not affected by shuffling). In other words, the author’s proposal would only inform why we observe higher fractions of 3n exons, but not higher overall frequencies of exon skipping. While this seems to be the statement in the first paragraph of the manuscript, it is not clear towards the end whether the author is trying to link both steps. Third, there are three potential caveats to keep in mind in Patthy’s proposal when provided as an explanation for our first proposed step (i.e. that all metazoans have a higher frequency of frame-preserving exon skipping): (i) The vast majority of skipped exons overlap with disordered regions and avoid protein domains [8–10], therefore, those exons are unlikely to have originated by exon-shuffling from ‘symmetrical’ modules; the ‘classic’ idea that exon skipping is a way to combine protein modules is an elegant one, but largely not supported by transcriptomic data. (ii) If the effect of the symmetrical modules were widespread across animals, we would have expected all exons, not only the skipped ones, to show some 3n enrichment, particularly in non-bilaterians; this is not the case. (iii) We have previously shown that exon skipping occurs at similar frequency in ancient (paneukaryotic) than in animal-specific genes [11]. Therefore, although we have not formally tested this in our manuscript, the 3n enrichment is likely to be largely independent of the time of gene’s origin. In conclusion, while we agree that this is an interesting proposal worth exploring further, it needs a comprehensive data update to clarify the origin of the associated genome-wide patterns as well as to more specifically test the associated predictions with current transcriptomic data.

Review written by Xavier Grau-Bové and Manuel Irimia.

References [1] França GS, Cancherini D V, de Souza SJ. 2012. Evolutionary history of exon shuffling. Genetica 140: 249–57. [2] Srivastava M, Simakov O, chapman J, Fahey B, Gauthier MEA, Mitros T, Richards GS, Conaco C, Dacre M, Hellsten U, et al. 2010. The Amphimedon queenslandica genome and the evolution of animal complexity. Nature 466: 720–726. [3] Srivastava M, Begovic E, chapman J, Putnam NH, Hellsten U, Kawashima T, Kuo a, Mitros T, Salamov a, carpenter ML, et al. 2008. The Trichoplax genome and the nature of placozoans. Nature 454: 955–960. [4] Putnam NH, Srivastava M, Hellsten U, dirks B, chapman J, Salamov a, Terry a, Shapiro H, Lindquist E, Kapitonov V V, et al. 2007. Sea anemone genome reveals ancestral eumetazoan gene repertoire and genomic organization. Science (80-) 317: 86–94. [5] King N, Westbrook MJ, young SL, Kuo a, Abedin M, chapman J, Fairclough S, Hellsten U, Isogai Y, Letunic I, et al. 2008. The genome of the choanoflagellate Monosiga brevicollis and the origin of metazoans. Nature 451: 783–8. [6] Suga H, Chen Z, de Mendoza a, Sebé-Pedrós a, Brown MW, Kramer E, Carr M, Kerner P, Vervoort M, Sánchez-pons N, et al. 2013. The Capsaspora genome reveals a complex unicellular prehistory of animals. Nat Commun 4: 2325. [7] Grau-Bové X, Torruella G, Donachie S, Suga H, Leonard G, Richards TA, Ruiz-Trillo I. 2017. Dynamics of genomic innovation in the unicellular ancestry of animals. Elife 6: Pii: e26036. [8] Romero PR, Zaidi S, fang YY, Uversky VN, Radivojac P, Oldfield CJ, Cortese MS, Sickmeier M, LeGall T, Obradovic Z, dunker AK. 2006. Alternative splicing in concert with protein intrinsic disorder enables increased functional diversity in multicellular organisms. Proc Natl Acad Sci U S a 103:8390–5. [9] Ellis JD, barrios-Rodiles M, Colak R, Irimia M, Kim T, Calarco JA, Wang X, Pan Q, O’Hanlon D, Kim PM, Wrana JL, Blencowe BJ. 2012. Tissue-specific alternative splicing remodels protein-protein interaction networks. Mol cell 46:884–92. [10] Buljan M, Chalancon G, Eustermann S, Wagner GP, Fuxreiter M, Bateman a, Babu MM. 2012. Tissue-specific splicing of disordered segments that embed binding motifs rewires protein interaction networks. Mol cell 46:871–83. [11] Irimia M, Rukov JL, penny D, Roy SW. 2007. Functional and evolutionary analysis of alternatively spliced genes is consistent with an early eukaryotic origin of alternative splicing. BMC Evol biol 4;7:188

### Reviewer’s report 2: Ashish Lal, National Cancer Institute, National Institute of Health, USA

## Reviewer’s comment

This is a nice discussion and suggestions on the Grau-Bové et al. paper that appeared recently in Genome Biology.

### Reviewer’s report 3: Erez Levanon, Bar-Ilan University, Israel

## Reviewer’s comment

In the opinion manuscript “Exon skipping-rich transcriptomes of animals reflect the significance of exon-shuffling in metazoan proteome evolution” the author offers an alternative explanation for the recently published observation that exon skipping in animals is more prevalent than in plants. The possible reason, the author claim, is that “multidomain proteins unique to metazoa and indispensable for metazoan type multicellularity were assembled by exon-shuffling from ‘symmetrical’ (i.e. 3n) modules, whereas this type of protein evolution played a minor role in other groups of eukaryotes, including plants”. While this theory is reasonable, the author should provide some experimental evidence to support it. E.g. showing that indeed alternatively spliced exons from multidomain proteins which are unique to metazoa and indispensable for metazoan type multicellularity are contributing the lion share of alternative spicing exons in animals.
